# Mild Encephalitis/Encephalopathy With a Reversible Splenial Lesion (MERS) in an Adult Following Influenza A Infection

**DOI:** 10.7759/cureus.82828

**Published:** 2025-04-23

**Authors:** Ahmed Malik, Sadia Sultana, Ramla Warsame, Ruju Smirtha Thillai, Uday Gollapinni

**Affiliations:** 1 Acute Internal Medicine, University Hospitals of North Midland, Royal Stoke University Hospital, Stoke on Trent, GBR

**Keywords:** corpus callosum, cytokine injury, influenza a, mers, mri, neurological deficits, splenial lesion, viral encephalopathy

## Abstract

Mild encephalitis/encephalopathy with a reversible splenial lesion (MERS) is a rare clinical-radiological entity predominantly reported in pediatric populations, with limited cases in adults. We present a case of a 27-year-old female who developed transient neurological deficits following influenza A infection. She presented with severe headache, neck stiffness, and limb weakness with sensory deficits. Initial suspicion of meningoencephalitis prompted treatment with intravenous antibiotics and antivirals. However, an MRI revealed a hyperintense splenial lesion in the corpus callosum (SCC), supporting a diagnosis of MERS. The patient achieved full recovery without corticosteroids, reinforcing the self-limiting nature of this condition. This case highlights the diagnostic challenges, imaging findings, and management considerations of MERS, underscoring the importance of clinical awareness to avoid unnecessary interventions in adult cases.

## Introduction

Influenza is an acute viral infection primarily transmitted via respiratory droplets and contaminated surfaces. Of the four influenza subtypes (A, B, C, and D), influenza A and B are responsible for seasonal epidemics, while influenza C causes mild illness, and influenza D primarily affects cattle, with no known human infections [1]. The severity of influenza varies from mild upper respiratory symptoms to life-threatening complications, including pneumonia, renal failure, and neurological manifestations such as Guillain-Barré syndrome (GBS) and encephalopathy [2]. Mild encephalitis/encephalopathy with a reversible splenial lesion (MERS) is a transient neurological syndrome associated with various infections, including influenza. It is characterized by MRI findings of hyperintensity in the SCC and is more commonly reported in pediatric populations [3]. This report describes an adult case of MERS secondary to influenza A, highlighting diagnostic challenges, pathophysiology, and management considerations [4].

## Case presentation

A 27-year-old female was referred by a general practitioner to the emergency department with suspected meningitis. She presented with a cough-producing green sputum, rigors, severe headache, neck stiffness, progressive limb weakness, and sensory deficits. She also reported a 24-hour history of diarrhea and vomiting. Her medical history included depression, and she had received all childhood vaccinations except the meningitis vaccine. On examination, she was fully conscious with a Glasgow Coma Scale score of 15/15 and demonstrated reduced muscle power: 3/5 in the upper limbs and 2/5 in the lower limbs. Sensory deficits in glove-and-stocking distribution were noted, with diminished pinprick sensation and impaired temperature perception. 

Investigations

Laboratory findings were largely unremarkable. Renal function tests, liver function tests, vitamin B12, folate, and blood glucose levels were all within normal ranges. However, the patient had low inorganic phosphate (0.4 mmol/L) and an elevated C-reactive protein (CRP: 47.5 mg/L). The low phosphate was corrected with an intravenous infusion.

Due to the initial suspicion of meningoencephalitis, intravenous ceftriaxone and acyclovir were administered according to hospital protocol, along with supportive management including intravenous fluids. A lumbar puncture was also planned. A rapid antigen test confirmed an influenza A infection.

Although her neurological symptoms initially resolved, they recurred later that night, accompanied by photophobia and loss of vibration sensation in both great toes. Given the evolving neurological picture, an urgent MRI of the brain and spine was performed, revealing hyperintensity in the SCC with diffusion restriction, consistent with MERS type 1 [1,3].

**Figure 1:**
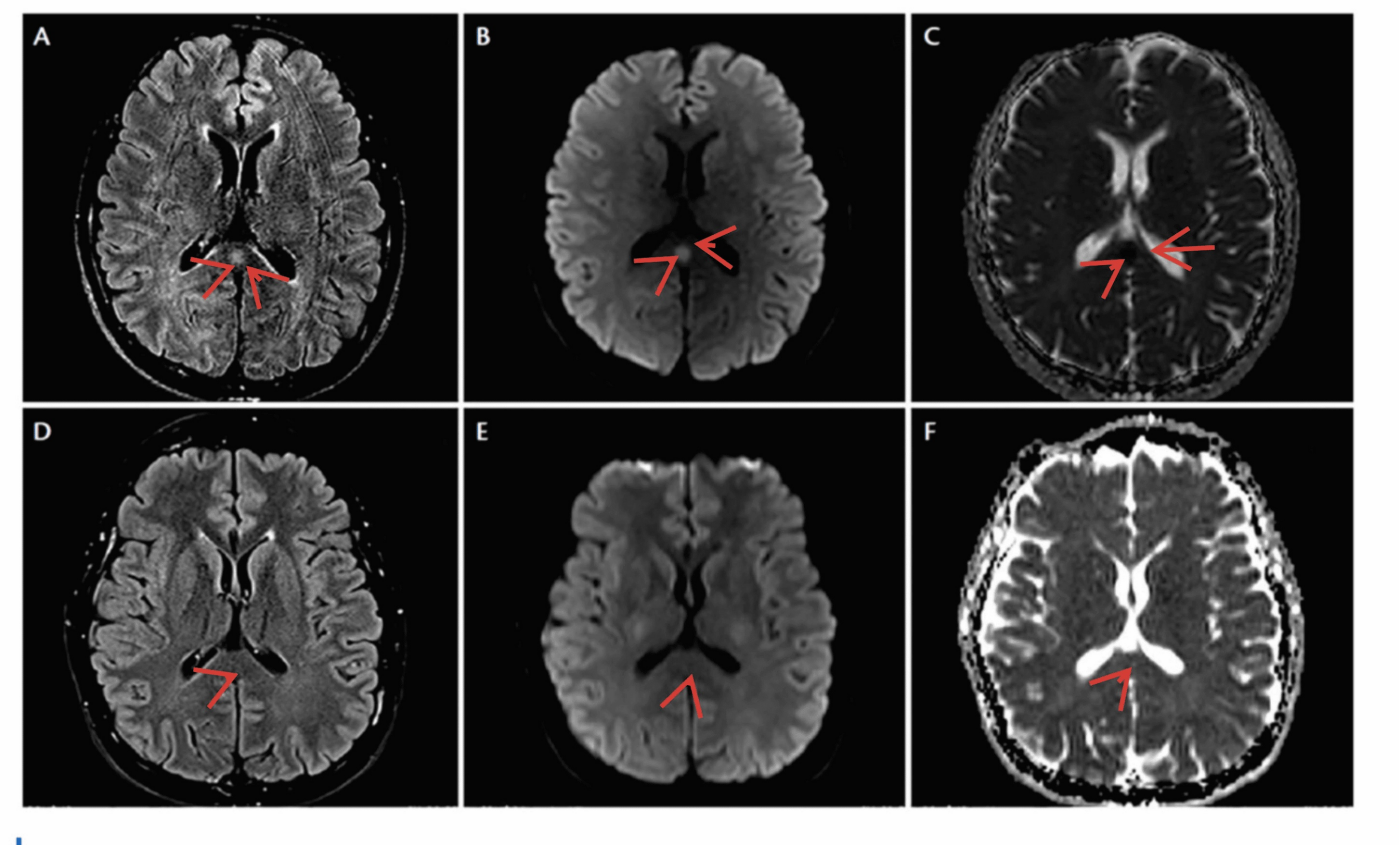
MRI of the head Brain MRI shows a small, ovoid area of increased signal intensity in the center of the splenium of the corpus callosum on axial FLAIR (A) and DWI (B), with corresponding diffusion restriction on the ADC image (C). Repeat FLAIR (D), DWI (E), and ADC (F) imaging performed at three months demonstrate complete resolution of the previously noted abnormal signals in the splenium. FLAIR, fluid-attenuated inversion recovery; DWI, diffusion-weighted imaging; ADC, apparent diffusion coefficient

The Neurology team was consulted, and the patient was reviewed by an Acute Medicine Consultant, who diagnosed influenza A infection with MERS type 1. Antibiotics and acyclovir were discontinued, and oseltamivir was initiated. The Neurology Consultant concurred with the management plan and advised that a lumbar puncture was unnecessary. Relevant blood test results are presented in Table 1 [1], and imaging findings from the MRI of the head are shown in Figure 1 [1-3].

**Table 1:** Blood test

Investigation	Result	Normal reference range
Hb	126 g/L	115-165 g/L
WBC	3.1	4-11
PLT	292	150-450
B12	261	200-900 pg/mL
Influenza A	Detected	-
Influenza B	Not detected	-
Covid-19	Not detected	-
Mg	0.77	0.70-1.0 mmol/L
Ca	2.33	2.2-2.6 mmol/L
Albumin	38	35-50 g/L
Phosphate	0.4	0.80-1.5 mmol/L
ALP	57	30-130 U/L
Na	137	133-146 mmol/L
K	3.8	3.5-5.3 mmol/L
Urea	3.4	2.5-7.8 mmol/L
ALT	15	0-48 IU/L
AST	19	0-35
Total bilirubin	7	0-21 umol/L
Creatinine	47	53-97.2 mmol/L
eGFR	>90	>90
CRP	47.5	0-5 mg/L
Blood glucose	5.1	4-7 mmol/L

## Discussion

MERS is a rare clinico-radiological syndrome characterized by transient neurological symptoms and reversible lesions in the SCC seen on MRI. It is predominantly reported in pediatric populations, with limited cases documented in adults [1].

The diagnosis of MERS is based on five criteria: 1) onset of neurological symptoms within one week of fever, 2) complete resolution of symptoms within 10 days, 3) high signal intensity in the SCC (MERS type 1), 4) diffuse involvement of the corpus callosum or white matter (MERS type 2), 5) resolution of lesions without residual changes on imaging [2].

Our patient fulfilled all diagnostic criteria for MERS type 1; follow-up MRI demonstrated complete resolution of the lesion. Literature suggests that repeat imaging may not be necessary if there is clear clinical improvement.

The pathophysiology of MERS is believed to involve cytokine-mediated disruption of the blood-brain barrier and transient intramyelinic edema, rather than direct viral invasion. Elevated levels of interleukins IL-6 and IL-10 have been implicated in influenza-associated encephalopathy and correlate with disease severity. The SCC’s dense fiber composition and high metabolic demand may predispose it to transient excitotoxic injury [3].

Given its broad differential diagnosis, including ischemia, demyelination, posterior reversible encephalopathy syndrome (PRES), metabolic encephalopathies, and infections, early imaging and clinical correlation are crucial. Cerebrospinal fluid (CSF) analysis typically reveals no pleocytosis, distinguishing MERS from infectious encephalitis. In our case, lumbar puncture was not performed due to the patient’s rapid neurological recovery, which represents a limitation in excluding alternative diagnoses [4].

Management of MERS is primarily supportive, with most cases resolving spontaneously. Although corticosteroids have been used in select cases, their benefit remains unproven [5]. Our patient recovered fully without corticosteroid therapy, supporting a conservative management approach. While oseltamivir-induced neurotoxicity has been reported, it was unlikely in this case, as the patient’s neurological symptoms predated the initiation of antiviral therapy.

## Conclusions

MERS is a rare but important consideration in patients presenting with neurological deficits following viral infections like influenza A. This case highlights the diagnostic challenges of MERS in adults, where symptoms may mimic more common conditions such as meningoencephalitis, GBS, and myelitis. MRI remains the cornerstone of diagnosis, with the presence of a hyperintense lesion in the splenium of the corpus callosum being pathognomonic. As demonstrated in this case, MERS is self-limiting, and most patients recover without the need for corticosteroid therapy. Early recognition, appropriate imaging, and supportive management are crucial in ensuring favorable outcomes while avoiding unnecessary interventions. This case emphasizes the need for heightened awareness of MERS in adult populations, particularly in the context of post-influenza complications.
